# Glycolysis Inhibition of Autophagy Drives Malignancy in Ovarian Cancer: Exacerbation by IL-6 and Attenuation by Resveratrol

**DOI:** 10.3390/ijms24021723

**Published:** 2023-01-15

**Authors:** Chiara Vidoni, Alessandra Ferraresi, Letizia Vallino, Amreen Salwa, Ji Hee Ha, Christian Seca, Beatrice Garavaglia, Danny N. Dhanasekaran, Ciro Isidoro

**Affiliations:** 1 Laboratory of Molecular Pathology, Department of Health Sciences, Università del Piemonte Orientale “A. Avogadro”, Via Solaroli 17, 28100 Novara, Italy; 2 Stephenson Cancer Center, The University of Oklahoma Health Sciences Center, Oklahoma City, OK 73104, USA

**Keywords:** Warburg effect, glycolysis, ovarian cancer, hexokinase 2, nutraceuticals, pro-inflammatory cytokines, autophagy, cell migration, overall survival, personalized cancer therapy

## Abstract

Cancer cells drive the glycolytic process towards the fermentation of pyruvate into lactate even in the presence of oxygen and functioning mitochondria, a phenomenon known as the “Warburg effect”. Although not energetically efficient, glycolysis allows the cancer cell to synthesize the metabolites needed for cell duplication. Autophagy, a macromolecular degradation process, limits cell mass accumulation and opposes to cell proliferation as well as to cell migration. Cancer cells corrupt cancer-associated fibroblasts to release pro-inflammatory cytokines, which in turn promote glycolysis and support the metastatic dissemination of cancer cells. In mimicking in vitro this condition, we show that IL-6 promotes ovarian cancer cell migration only in the presence of glycolysis. The nutraceutical resveratrol (RV) counteracts glucose uptake and metabolism, reduces the production of reactive oxygen species consequent to excessive glycolysis, rescues the mitochondrial functional activity, and stimulates autophagy. Consistently, the lack of glucose as well as its metabolically inert analogue 2-deoxy-D-glucose (2-DG), which inhibits hexokinase 2 (HK2), trigger autophagy through mTOR inhibition, and prevents IL-6-induced cell migration. Of clinical relevance, bioinformatic analysis of The Cancer Genome Atlas dataset revealed that ovarian cancer patients bearing mutated *TP53* with low expression of glycolytic markers and IL-6 receptor, together with markers of active autophagy, display a longer overall survival and are more responsive to platinum therapy. Taken together, our findings demonstrate that RV can counteract IL-6-promoted ovarian cancer progression by rescuing glycolysis-mediated inhibition of autophagy and support the view that targeting Warburg metabolism can be an effective strategy to limit the risk for cancer metastasis.

## 1. Introduction

Non-malignant cells physiologically achieve most of their energy from mitochondrial oxidative phosphorylation producing approximately 36 molecules of ATP per one molecule of glucose, while the transformation of pyruvic acid into lactic acid yields just two molecules of ATP [1]. In the presence of oxygen, normal cells utilize oxidative phosphorylation as metabolic source of energy production, whereas in the absence of oxygen, the pyruvate is predominantly metabolized into lactate [2]. However, proliferating cancer cells must exploit glucose as the main carbon source for both ATP and macromolecular biosynthesis [3,4]. Therefore, regardless of the availability of oxygen, tumor cells obtain energy and metabolites from glucose metabolism through glycolysis (a process known as “Warburg effect”), with high production of lactic acid [5]. The Warburg effect is an adaptive mechanism regulated by oncogene activation and tumor suppressor gene inactivation that support the tumorigenic process [6]. Since the Warburg effect is not energetically efficient for the tumor, cancer cells increase the glucose intake and the glycolytic rate. Accordingly, members of the glucose transporters (GLUTs) family, such as GLUT1 and GLUT4, are over-expressed in several types of cancers, including ovarian cancer, and this correlates with poor prognosis [7,8,9]. Additionally, the secretion of lactate leads to the acidification of the tumor microenvironment, which contributes to the degradation of extracellular matrix, tumor invasion, and metastasis [10].

Interleukin-6 (IL-6) is a pleiotropic cytokine that regulates several processes, such as cell metabolism, inflammation, and immune response [11]. IL-6 is secreted not only by cancer cells, but also from stromal cells in the tumor microenvironment [12,13]. IL-6 accumulates in the serum and ascitic liquid of ovarian cancer patients [14], correlating with metastasis and poor clinical outcome [15]. We demonstrated that IL-6 positively impinges on ovarian cancer cell proliferation and motility via epigenetic down-regulation of autophagy [16,17]. We also showed that the nutraceutical resveratrol (RV) contrasts the oncogenic activities of IL-6 in the tumor microenvironment, acting as a potent inducer of autophagy and modulator of the transcriptome and epigenome [16,17,18,19,20].

Here, we report that IL-6 induces ovarian cancer cell migration only in the presence of glucose, and this effect is hampered by RV. We found that RV mimics a glucose deprivation condition, reducing the plasma membrane expression of GLUT1 and the glucose intake. IL-6-induced cell migration is paralleled by inhibition of autophagy at the migration front, a phenomenon not occurring when glycolysis is inhibited either by the metabolically inert glucose analogue 2-DG or the LDH inhibitor sodium oxamate. Interrogation of The Cancer Genome Atlas (TCGA) revealed that patients bearing *TP53* mutated ovarian cancer with low expression of glycolytic markers and IL-6 receptor together with an efficient autophagy machinery survive longer and are more responsive to the platinum therapy. Overall, the present data demonstrate that glycolytic and autophagy markers are good prognostic indicators and that targeting cancer cell metabolism can be an effective strategy for reducing the risk of metastasis and improving ovarian cancer patients’ survival.

## 2. Results

### 2.1. IL-6-Induced Ovarian Cancer Cell Migration Relies on Glucose Metabolism

**Figure 1:**
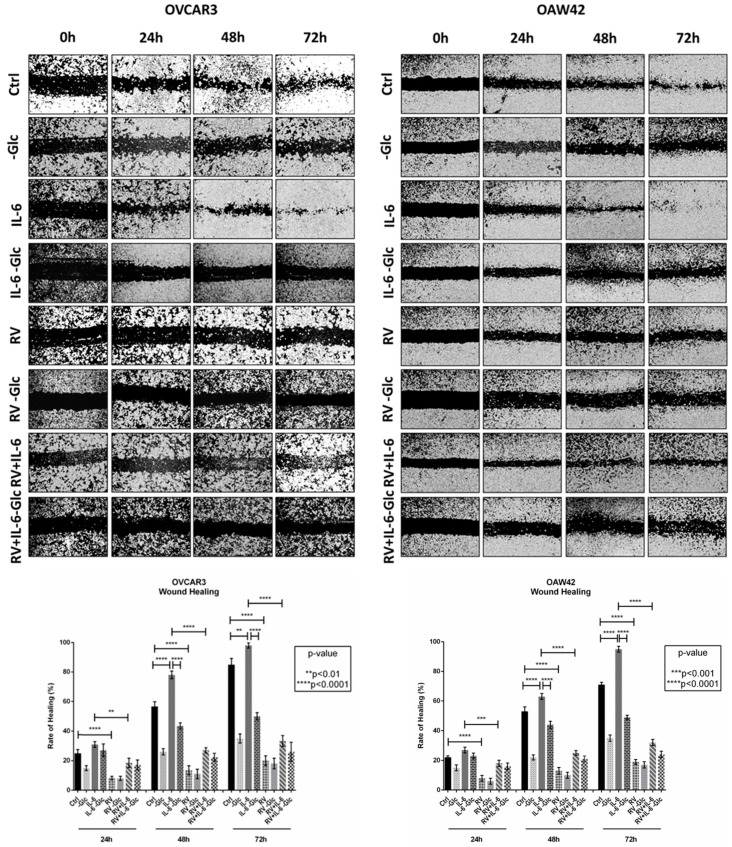
Resveratrol and glucose deprivation reduce IL-6-induced ovarian cancer cell migration. OVCAR3 and OAW42 cells were exposed to 100 µM resveratrol (RV) and 50 ng/mL IL-6, alone or in combination, in the presence or absence of glucose in culture medium. Medium was renewed every 24 h. The wound closure was monitored up to 72 h by using a phase-contrast microscope. The rate of healing (%) for each experimental time-point, resulting from cell motility, is calculated as the distance between the margins of the wound by using ImageJ software. Data represent the average  ±  SD calculated for three different fields per each condition.

To monitor ovarian cancer cell migration, we performed a wound healing scratch assay in OVCAR3, OAW42, and SKOV3 cell lines, representative of ovarian cancer heterogenicity and characterized by a different genetic background for oncogenes and tumor suppressor genes (Table 1). To assess the migratory capability of ovarian cancer cells in an inflammatory and glucose deprived microenvironment, OVCAR3 and OAW42 cells were treated with 50 ng/mL IL-6, 100 µM resveratrol (RV) or both in the presence or absence of glucose (Figure 1).

Both in OVCAR3 and OAW42 cells, in standard culture conditions, the wound was nearly completely healed (approximately 80%) by 72 h. The lack of glucose hampered cancer cell migration in both the cell lines. IL-6 promoted the healing only in the presence of glucose, markedly visible at 48 h. Conversely, the addition of RV to the culture medium counteracted cell motility even when the cells were challenged with the inflammatory cytokine, mimicking the trend observed in absence of glucose. Possibly, RV showed more effective than glucose deprivation in limiting cell motility even in the presence of IL-6.

However, IL-6 did not increase the motility of SKOV3 cells (Supplementary Figure S1), likely due to their low basal expression of IL-6 receptor (IL-6R) compared to that observed in OVCAR3 and OAW42 cells (about five-fold less) (Supplementary Figure S2). For these reasons, we excluded SKOV3 cell line from the next experiments.

**Figure 2:**
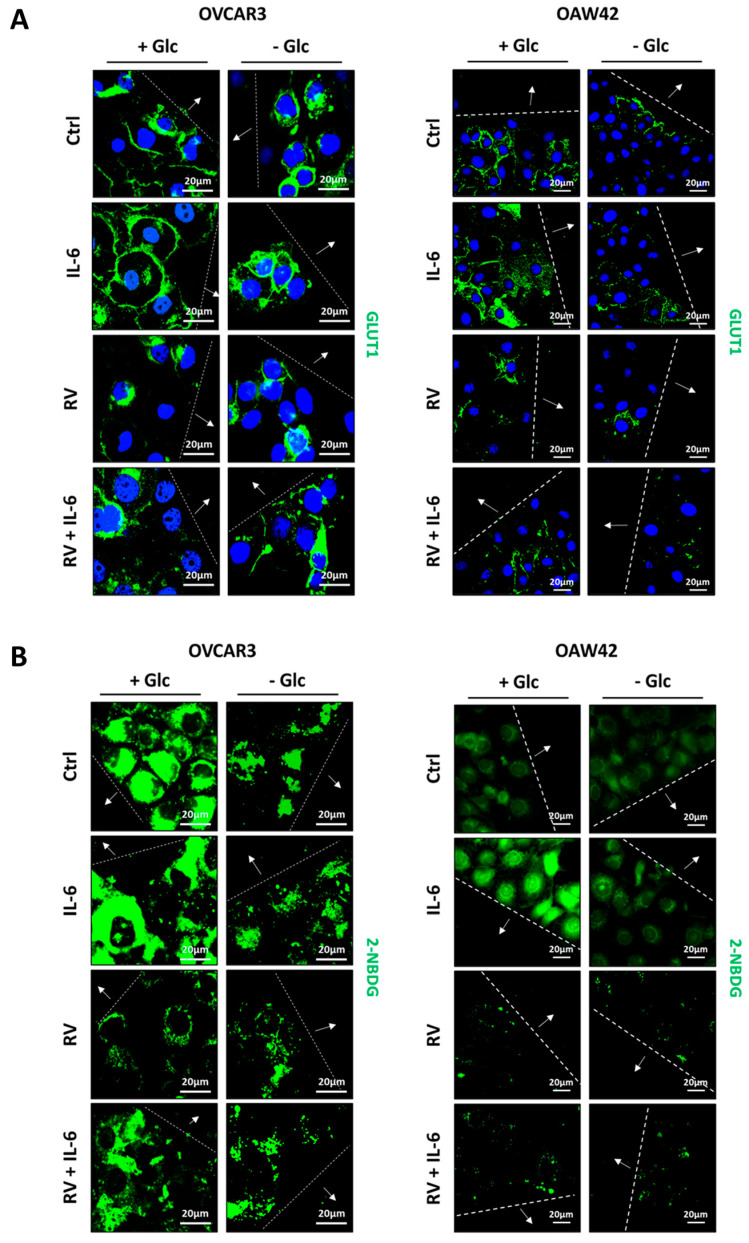
Resveratrol mimics glucose deprivation by preventing IL−6−induced-GLUT1 plasma membrane translocation and reducing glucose uptake. OVCAR3 and OAW42 cells were plated on coverslips and scratched to perform a wound. Cells were treated with 100 µM resveratrol (RV) and 50 ng/mL IL−6, alone or in combination, in the presence or absence of glucose in culture medium for 24 h. At the end, coverslips were (**A**) fixed and immunostained for GLUT1 (green); (**B**) incubated and stained with 2−NBDG. The cells were photographed under the fluorescence microscopy. Scale bar = 20 μm; magnification = 63×. Data are representative of different fields per each condition. The wound is marked by dotted lines and the arrows point to the migration front.

Given the role of glucose as main source of energy in cancer metabolism, we performed two different assays to evaluate the glucose uptake capability. First, we analyzed by immunofluorescence the localization of GLUT1, a specific membrane carrier that mediates glucose internalization, focusing on the cells at the migration front. We observed that IL-6 promoted the translocation of GLUT1 to the plasma membrane only if glucose was available. Interestingly, RV hampered this effect by keeping GLUT1 in a perinuclear localization, also in the presence of IL-6, displaying the same pattern of GLUT1 localization observed in the glucose-deprived cells (Figure 2A). Next, we monitored the uptake of a fluorescent glucose analogue, 2-NBDG, that acts as a competitor of glucose for the same membrane transporter of the GLUTs family (Figure 2B). In standard culture condition, OVCAR3 cells internalized a higher quantity of glucose compared OAW42 cells. Interestingly, IL-6-treated cells at the migration front displayed a high 2-NBDG uptake. Again, this trend was strongly reduced by RV, resembling glucose deprivation.

**Figure 3:**
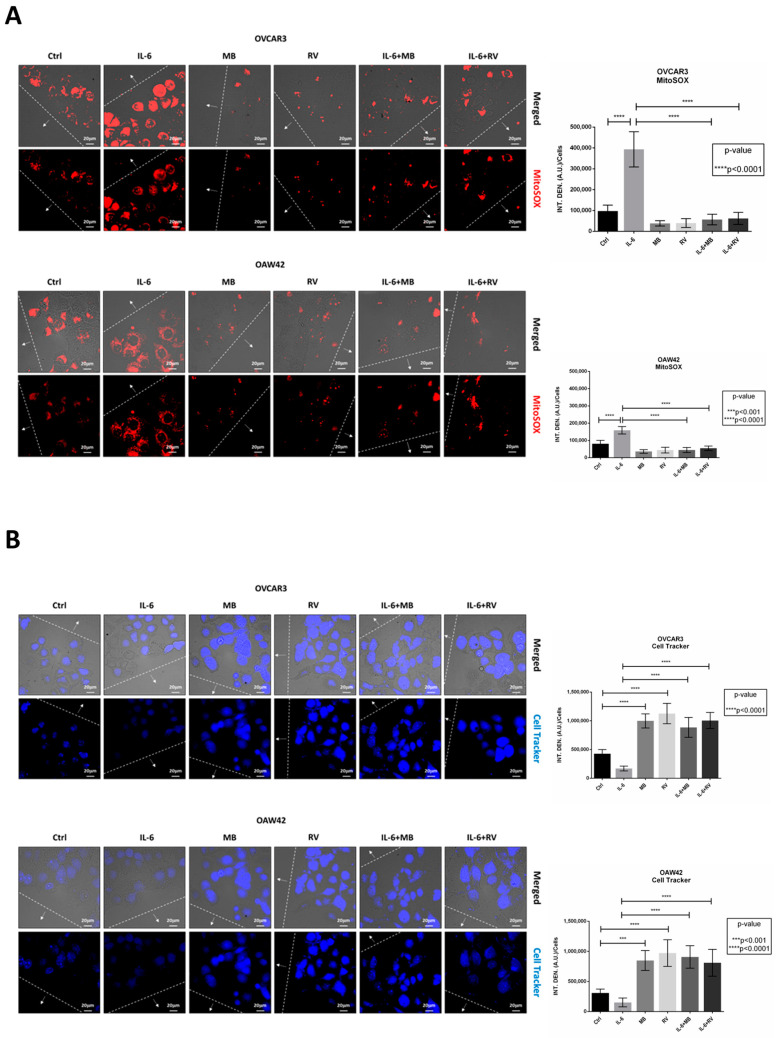
Resveratrol decreases anion superoxide levels and restores mitochondrial activity. OVCAR3 and OAW42 cells were plated on coverslips and treated with 100 μM RV and 10 μM MB, in presence or absence of 50 ng/mL IL-6 for 24 h. Cells were labelled with (**A**) MitoSOX™ and (**B**) Cell Tracker™ Blue fluorescent dyes. Coverslips were directly acquired at the fluorescent microscope. Scale bar = 20 μm; magnification = 63×. Data are representative of different fields per each condition. The wound is marked by dotted lines and the arrows point to the migration front. Graphs report the quantification of the fluorescence intensity per cell.

Next, we employed the fluorescent dye MitoSOX™ to assess the production of mitochondrial anion superoxide arising from excessive glycolytic rate (Figure 3A). As negative control for reactive oxygen species (ROS) production, we introduced methylene blue (MB), a known stimulator of the mitochondrial respiration [22]. The enhanced glycolytic flux correlates with increased ROS levels [23]. The high glucose uptake induced by IL-6 associated with increased levels of ROS (red signal), was more evident in OVCAR3 cells. In a separate study, we showed that under this condition cell viability is however not affected [17]. MB contrasted the production of anion superoxide induced by IL-6, confirming that these ROS arose from overwhelming the respiratory chain caused by increased glycolytic flux (Figure 3A). Worthy of note, RV also significantly reduced the generation of ROS even in the cells challenged with IL-6, mimicking the phenotype induced by MB. Given that RV inhibits the glucose uptake, this fact further confirms that overproduction of anion superoxide is a consequence of increased glucose metabolism.

In parallel, cells were labelled with Cell Tracker™ Blue dye, which fluorescence allows to monitor the functional mitochondrial activity (Figure 3B). In line with the previous results, IL-6-treated cells showed a decrease in blue fluorescence intensity compared to that of the controls, whereas RV significantly increased the Cell Tracker™ fluorescence even in the presence of IL-6. A similar effect was elicited by MB. Taken together, these data confirm that reducing the glycolytic flux benefits mitochondrial functioning.

### 2.2. High Expression of Glycolytic Markers Predicts Poor Prognosis in Ovarian Cancer Patients

We analyzed the clinical database (TCGA and Kaplan–Meier plotter) focusing on the individual prognostic value of the following glycolytic markers for ovarian cancer: (i) *SLC2A1* (GLUT1 gene), the carrier responsible for glucose internalization into the cells; (ii) *HK2*, the key enzyme for the rate-limiting step of glycolysis; and (iii) *SLC16A4* (MCT4 gene), the transporter of monocarboxylates mainly involved in the efflux of lactate deriving from glycolysis.

**Figure 4:**
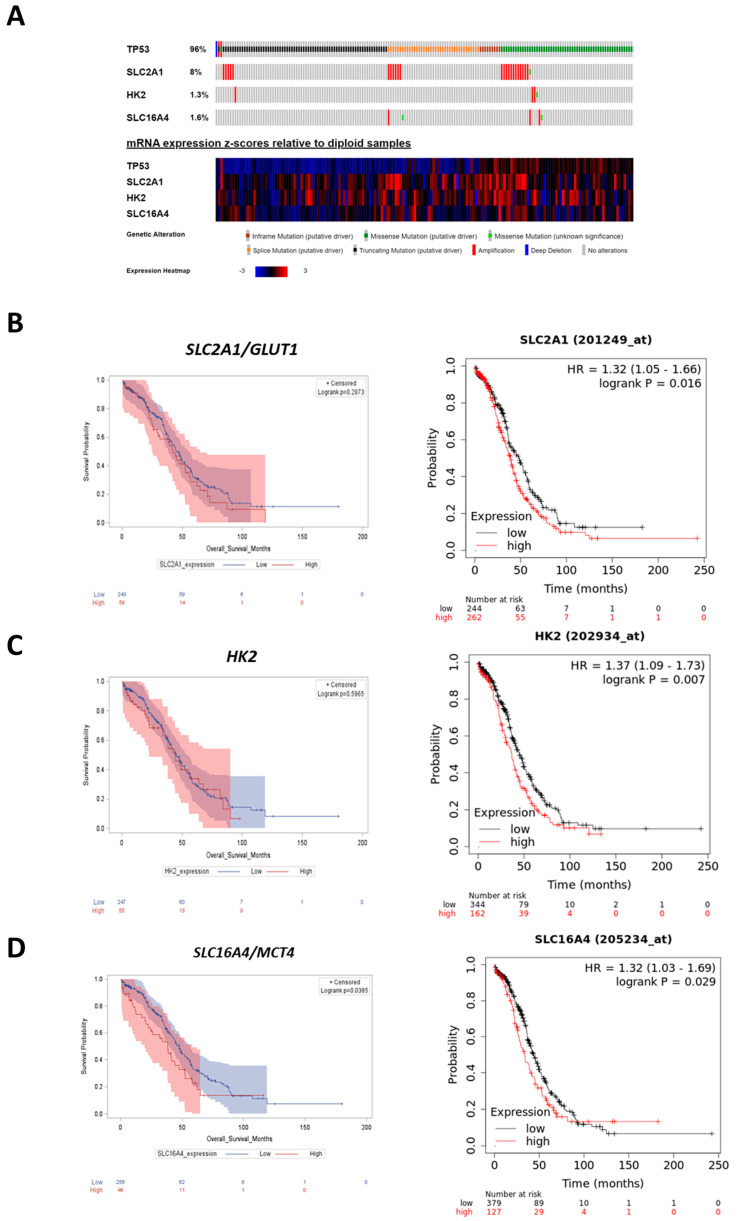
Patients with low expression of glycolytic markers have a longer overall survival. (**A**) The oncoprint of somatic mutations shows the genetic alterations and mRNA expression levels of *TP53*, *SLC2A1*, *HK2*, and *SLC16A4* in 316 ovarian serous cystadenocarcinoma patients (TCGA, Nature 2011). (**B**–**D**) Bioinformatic analysis performed on TCGA database (cohort n = 303) and Kaplan−Meier plotter (cohort n = 506) ovarian cancer patients. The overall survival curves were obtained based on *SLC2A1* (**B**), *HK2* (**C**), and *SLC16A4* (**D**) mRNA expression (high vs. low). The log−rank test has been used to determine the statistical significance. The *p*-value ≤ 0.05 was considered as significant.

Since most ovarian cancer patients bear a tumor with mutated *TP53* (96% of the cases) (Figure 4A), we restricted our analysis to those group of patients (TCGA (n = 303) and Kaplan–Meier plotter (n = 506)). As shown in Figure 4B–D, patients with low expression of *SLC2A1*, *HK2*, and *SLC16A4* have a better prognosis, in terms of longer overall survival, compared to the high expressor ones.

The clinical information of TCGA patients’ cohort together with the above results prompted us to select the OVCAR3 cell line for the next experiments. In fact, these cells express mutant *TP53* (R248Q) (Table 1) and can be considered a cellular model representative of the majority of ovarian cancer patients bearing mutations in *TP53* sequence (96% of the cases reported in TCGA patients’ cohort) (Figure 4A). Moreover, OVCAR3 cells show the highest migratory capability in basal condition compared to that of OAW42 cells (85% vs. 71%) (Figure 1) and display a more pronounced glucose-addicted phenotype (Figure 2B). OVCAR3 cells express mutant *PTEN* and *TP53*, resulting in a marked glycolytic phenotype due to the constitutive localization of the glucose transporters on the plasma membrane [21,24,25,26]. On the other hand, OAW42 cells express very low level of PTEN protein because of the methylated status of both the alleles, but present wild-type *TP53* that partially attenuates the Warburg effect [21].

### 2.3. Antagonizing Glucose Metabolism Hampers IL-6-Induced Ovarian Cancer Cell Migration

To confirm that IL-6 enhancement of ovarian cancer cell migration depends on glucose metabolism, we performed a wound healing scratch assay in the presence of 2-deoxy-D-glucose (2-DG) or sodium oxamate, which inhibit upstream and downstream the glycolytic pathway, respectively. 2-DG is a metabolically inert glucose analogue that inhibits glycolysis by inhibiting hexokinase 2 (HK2), while oxamate is a structural analog of pyruvate that inhibits the lactate dehydrogenase (LDH).

**Figure 5:**
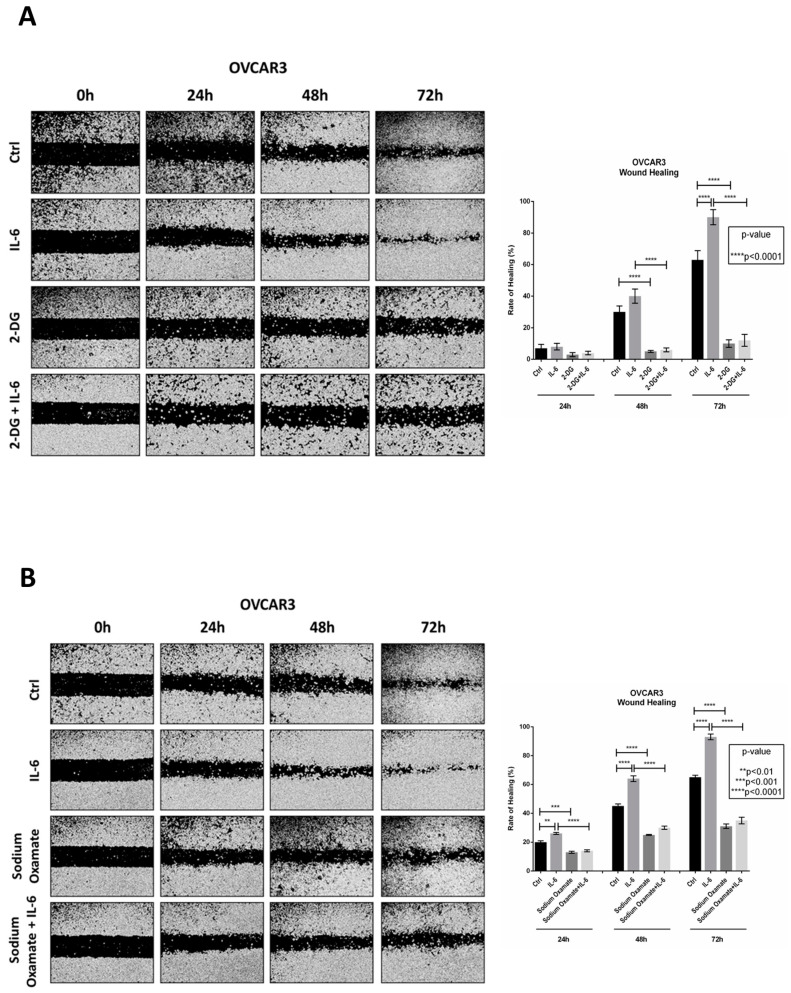
Inhibition of glucose metabolism impairs IL-6-induced ovarian cancer cell migration. OVCAR3 cells were exposed to (**A**) 20 mM 2-DG or (**B**) 20 mM sodium oxamate, alone or in combination with 50 ng/mL IL-6. Medium was renewed every 24 h. The wound closure was monitored up to 72 h by using a phase-contrast microscope. The rate of healing (%) for each experimental time-point was created by using ImageJ software. Data represent the average  ±  SD calculated for three different fields per each condition.

In agreement with the previous data, IL-6 strongly promoted cancer cell migration, causing the complete closure of the wound after 72 h. When 2-DG was added to the culture medium, the migration capability of OVCAR3 cells was strongly reduced (about 80%). Notably, the addition of IL-6 to 2-DG-treated cells failed to promote cell motility (Figure 5A). We observed a similar effect in the cells exposed to sodium oxamate, in which the rate of healing was around 30% even in the presence of IL-6, yet the effect was less pronounced (about 2-fold) compared to that of 2-DG (Figure 5B).

We have previously shown that autophagy counteracts cell migration in different tumor models [16,19,27,28]. HK2 represents the hub between energy production and energy conservation by triggering glycolysis or autophagy depending on the cellular energetic status and availability of glucose. In glucose-rich conditions, HK2 phosphorylates glucose to glucose-6-phosphate, promoting the glycolytic process, thus limiting autophagy. Conversely, under glucose deprivation, HK2 binds to and sequesters mTORC1 in the cytoplasm. When mTOR is detached from the lysosomal membrane, it cannot inhibit autophagy, which then is induced for maintaining cell homeostasis [29].

**Figure 6:**
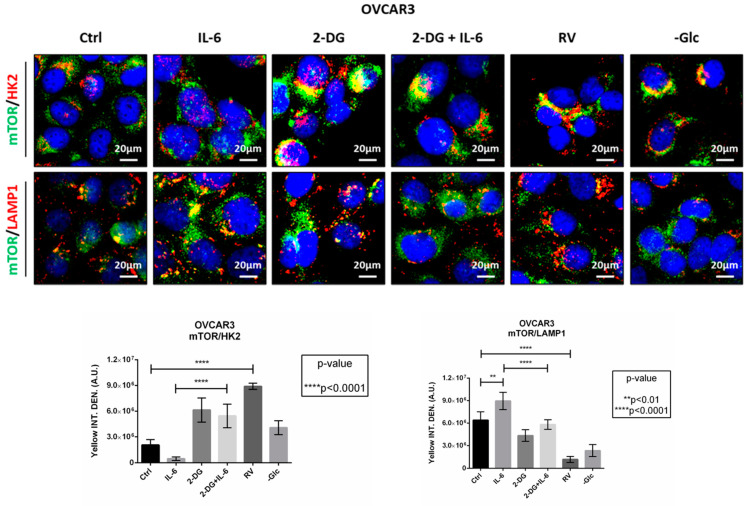
HK2 acts as the molecular switch controlling glucose metabolism and autophagy. OVCAR3 cells were plated on coverslips and treated as indicated for 24 h. Thereafter cells were fixed and double-stained for mTOR (green)/HK2 (red) or mTOR (green)/LAMP1 (red). Nuclei were stained with DAPI. The images were acquired at the fluorescence microscope. Scale bar = 20 μm; magnification = 63×. Data are representative of different fields per each condition. Graphs show the quantification of the average yellow fluorescence density (representing the co-localization between mTOR/HK2 and mTOR/LAMP1).

To investigate the cross-talk between glycolysis and autophagy, we performed two immunofluorescences double-staining for mTOR/HK2 and mTOR/LAMP1 (Figure 6). In control condition, mTOR was partially located on the lysosome membrane, and the addition of IL-6 further increased the co-localization of mTOR with the lysosomal marker LAMP1 (yellow signal), suggesting that in these cells mTOR is active and autophagy is inhibited. Conversely, in 2-DG-treated cells we observed the detachment of mTOR from the lysosome and an increased co-localization of mTOR with HK2, even in the presence of IL-6. The addition of RV to the culture medium exhibited a similar trend to that observed in 2-DG-treated cells, both resembling the effects observed in glucose-starved cells.

### 2.4. Resveratrol Modulates the Expression of Genes Involved in Glucose Metabolism and Autophagy

**Figure 7:**
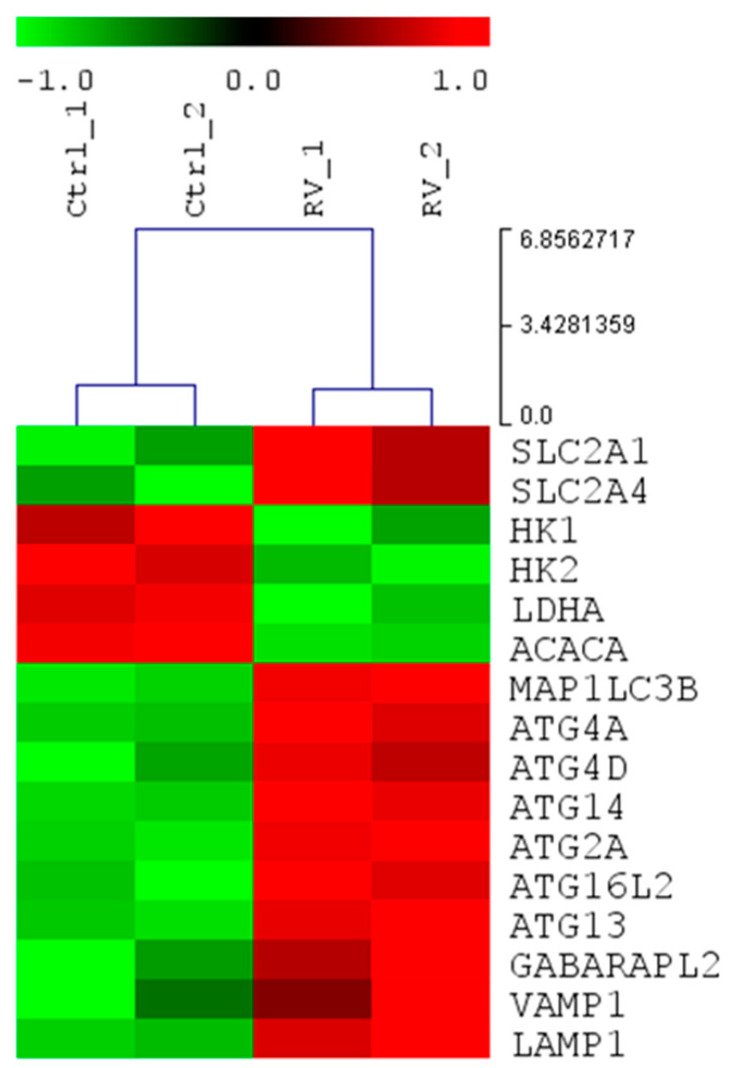
Resveratrol down-regulates glycolytic markers while up-regulates the expression of genes involved in autophagy machinery. Heatmap of the expression profiles of the differentially modulated genes belonging to glycolysis and autophagy pathways in OVCAR3 cells. The expression signature of untreated cells (first and second column) was compared to the transcriptome of OVCAR3 cells upon RV treatment (third and fourth column). Green and red colors represent down-regulation and up-regulation, respectively.

To dissect the impact of RV at transcriptomic level, we performed a whole mRNA expression profiling of OVCAR3 cells exposed to RV for 24 h. The heatmap reported in Figure 7 shows that RV decreases the mRNA expression of glycolytic markers, such as HK2 and LDHA, and ACC (acetyl-CoA carboxylase, *ACACA* gene), the substrate of AMPK kinase. At the same time, as a positive feedback mechanism, RV also induces the transcription of glucose transporters *SLC2A1* and *SLC2A4* genes. On the other hand, RV upregulates the transcripts of the autophagy machinery, in particular the genes involved in the induction complex (ATG13), assembly and formation of autophagosome, as well as in LC3 maturation and conjugation (i.e., ATG4, ATG16L), in the formation of lysosomes (LAMP1 and VAMP1), and in mitophagy (GABARAPL2).

### 2.5. IL-6 Inhibits Autophagy and Promotes Cancer Cell Migration Only if Glucose Is Metabolized

**Figure 8:**
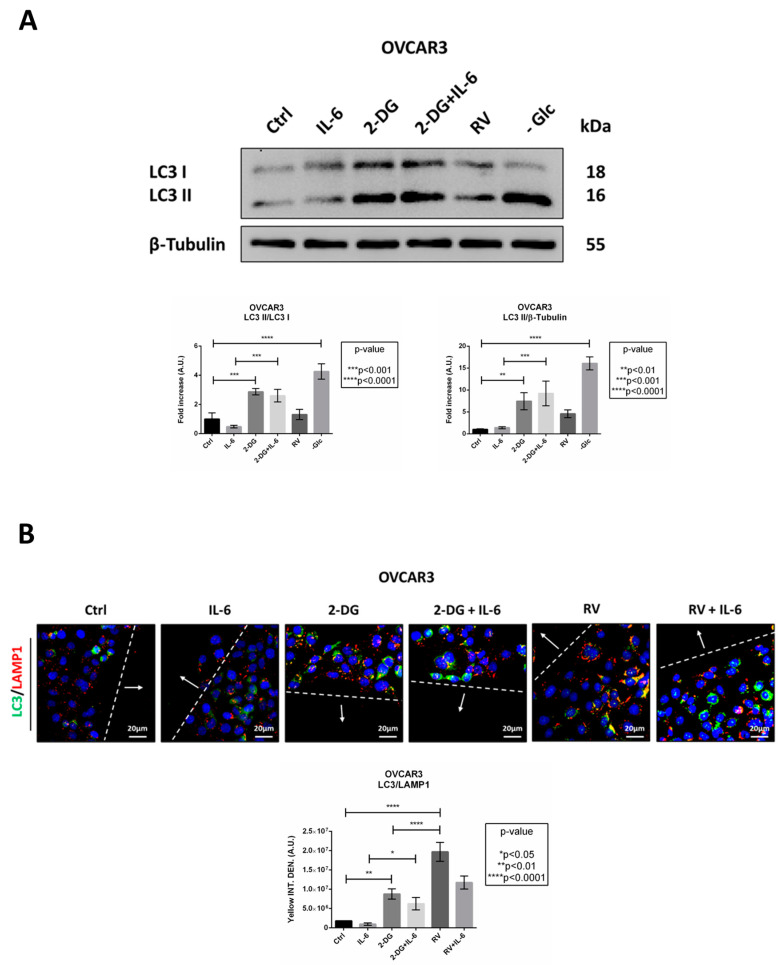
Resveratrol restores the IL-6-mediated inhibition of autophagy. OVCAR3 cells adherent on Petri dishes or coverslips were treated as indicated for 24 h. (**A**) Cell homogenates were analyzed by Western blotting for the expression of LC3. The filter was probed with β-tubulin as loading control. The Western blotting is representative of three replicates and densitometric data are reported in the graph. (**B**) OVCAR3 cells were treated as indicated for 24 h, fixed and double-stained for LC3 (green)/LAMP1 (red). Nuclei were stained with DAPI. The images were acquired in the proximity of the migration front at the fluorescence microscope. Scale bar = 20 μm; magnification = 63×. Data are representative of different fields per each condition. The wound is marked by doted lines and the arrows point to the migration front. The graph reports the quantification of the average yellow fluorescence density (representing the co-localization between LC3 and LAMP1).

To prove the involvement of autophagy, we performed a Western blotting analysis for LC3 expression (Figure 8A). IL-6-treated cells displayed an impairment of autophagy flux, as indicated by the lower LC3 II/LC3 I ratio compared to the control. The addition of 2-DG to the culture media resulted in a significant increase of autophagy flux as well as of LC3 II/β-Tubulin ratio, even in the presence of IL-6. Similarly, the treatment with RV or glucose starvation resulted in an enhanced autophagy flux and increased formation of autophagosomes, indicating that RV can reproduce the metabolic changes promoted by the inhibition of glucose metabolism.

To confirm the above findings, we monitored autophagy in the cells at the migration front by immunofluorescence double-staining of LC3 and LAMP1 (Figure 8B). IL-6-treated cells displayed a lower number of LC3-positive vacuoles as well as a decreased co-localization of LC3 and LAMP1. The addition of 2-DG resulted in autophagy induction, as indicated by the increase of LC3 fluorescence puncta as well as of the yellow signal (representing the autolysosomes). RV promoted the formation of LC3-positive spots and strongly induced autophagy in the cells at the migration front, mimicking the 2-DG condition. Taken together, these data support the view that either the glucose deprivation, the treatment with RV, or the incubation with 2-DG promotes autophagy, that in turn hampers ovarian cancer cell migration.

### 2.6. IL-6 and RV Oppositely Modulate the Expression of EMT and Autophagy Markers

During migration, the cells acquire a mesenchymal phenotype characterized by the switch in the expression of the adhesion membrane cadherin from epithelial-like (E) to neural-like (N) type. This change marks the epithelial to mesenchymal transition (EMT) process, which is governed by the transcription factor TWIST1, beside others. Autophagy regulates this phenoconversion participating in the degradation of EMT markers [19,27].

**Figure 9:**
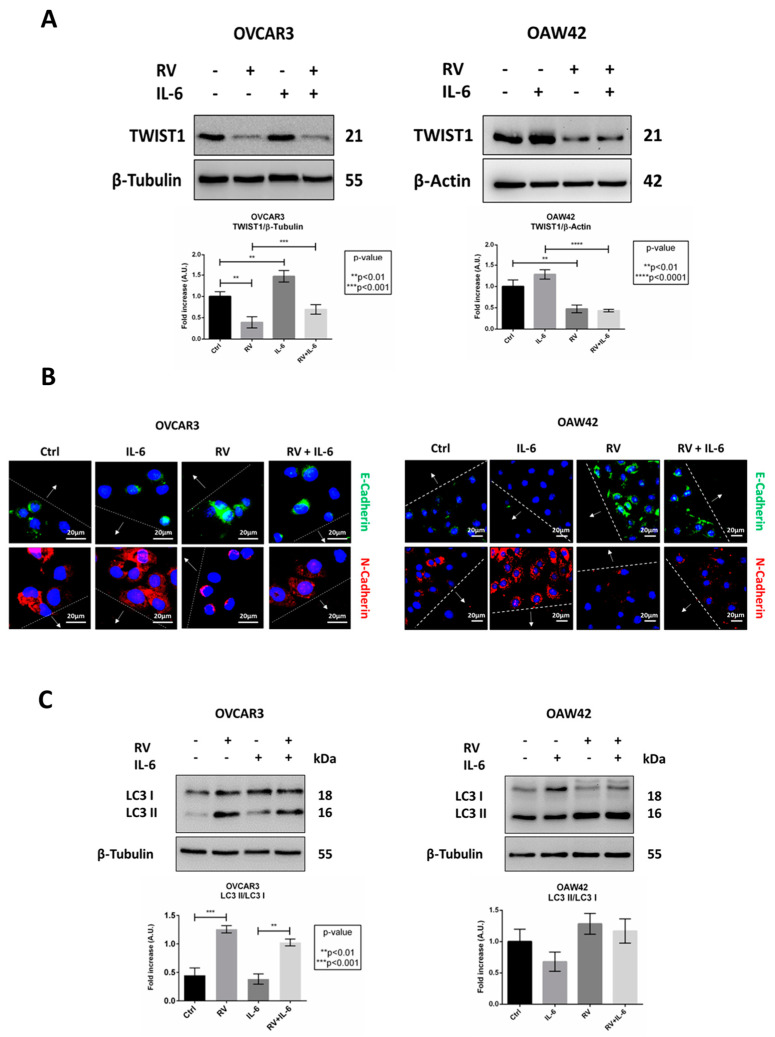
Resveratrol restores the epithelial phenotype and rescues the IL−6−mediated inhibition of autophagy. OVCAR3 and OAW42 cells adherent on Petri dishes or coverslips were treated as indicated for 24 h. (**A**) Cell homogenates were analyzed by Western blotting for the expression of TWIST1. The filter was probed with β−tubulin and β−actin as loading control. The Western blotting is representative of three replicates and densitometric data are reported in the graph. (**B**) OVCAR3 and OAW42 were fixed and stained for E-cadherin (green) and N-cadherin (red). Nuclei were stained with DAPI. The images were acquired in the proximity of the migration front at the fluorescence microscope. Scale bar = 20 μm; magnification = 63×. Data are representative of different fields per each condition. The wound is marked by dotted lines and the arrows point to the migration front. (**C**) Cell homogenates were analyzed by Western blotting for the expression of LC3. The filter was probed with β-tubulin as loading control. The Western blotting is representative of three replicates and densitometric data are reported in the graph.

Compared to the control condition, IL-6 increases the expression of TWIST1 (Figure 9A) and promotes the mesenchymal switch from E-cadherin to N-cadherin (Figure 9B), along with the inhibition of autophagosome maturation (Figure 9C). RV acts in an opposite manner, strongly reducing the expression of TWIST1 (Figure 9A), restoring the epithelial phenotype (Figure 9B), and promoting autophagosome formation (Figure 9C). These effects have been observed in both OVCAR3 and OAW42 cell lines.

### 2.7. Low Expression of IL6R, SLC2A1 (GLUT1) and HK2 with High MAP1LC3B Expression Correlates with Better Prognosis in TP53 Mutated Ovarian Cancer Patients

**Figure 10:**
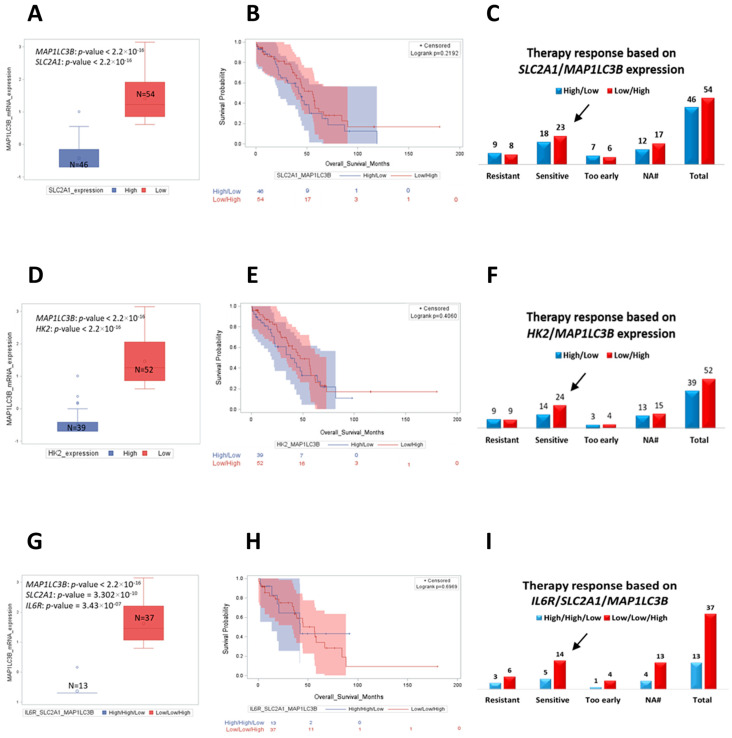
Patients with low expression of *SLC2A1*, *HK2*, and *IL6R* markers together with *MAP1LC3B* upregulation display a longer overall survival and are more responsive to platinum therapy. Analysis of the clinical outcome of ovarian cancer patients performed by interrogating TCGA bioportal (ovarian serous cystadenocarcinoma dataset, TCGA Nature 2011). Patients whose therapy response status was not available were classified in N/A group. Box plot showing the distribution of *MAP1LC3B* mRNA expression according to: (**A**) *SLC2A1* mRNA levels (high vs. low group); (**D**) *HK2* mRNA levels (high vs. low group); (**G**) high *IL6R*/high *SLC2A1*/low *MAP1LC3B* vs. low *IL6R*/low *SLC2A1*/high *MAP1LC3B* mRNA levels. Kaplan−Meier plot representing the overall survival status of patients stratified based on the differential expression of: (**B**) *SLC2A1* and *MAP1LC3B* (high/low vs. low/high), respectively; (**E**) *HK2* and *MAP1LC3B* (high/low vs. low/high), respectively; (**H**) *IL6R*, *SLC2A1*, and *MAP1LC3B* (high/high/low vs. low/low/high), respectively. Graphs reporting the response to platinum therapy based on the differential mRNA expression of: (**C**) *SLC2A1* and *MAP1LC3B*; (**F**) *HK2* and *MAP1LC3B*; (**I**) *IL6R*, *SLC2A1*, and *MAP1LC3B*. The histograms report the number of patients that were resistant, sensitive, or developed chemoresistance as soon as the chemotherapy treatment is administrated (too−early group).

Finally, to corroborate and further support the translational relevance of our findings, we interrogated the TCGA dataset searching for a link between glucose metabolism and autophagy with clinical outcome. We compared the overall survival of patients bearing opposite expression of *SLC2A1* and *HK2* with *MAP1LC3B* (autophagosome marker) and their responsiveness to platinum therapy. We found that the mRNA expression of *SLC2A1* and *HK2* were inversely correlated with that of *MAP1LC3B* (Figure 10A,D). In addition, we observed that patients with low expression of *SLC2A1* and *HK2* together with high expression of *MAP1LC3B* showed a better outcome in terms of overall survival (Figure 10B,E). Notably, the number of patients that were sensitive to platinum treatment was higher in the group with low levels of *SLC2A1* and *HK2* and high *MAP1LC3B* expression compared to the ones with high levels of glycolytic markers and downregulated autophagy (Figure 10C,F).

We observed that *MAP1LC3B* was inversely correlated with *IL6R* and *SLC2A1* mRNA expression in ovarian cancer patients TCGA data set (Figure 10G). Next, we compared the clinical outcome of patients with high expression of *IL6R* and *SLC2A1*, together with low *MAP1LC3B* with the ones with low *IL6R*, low *SLC2A1*, and high *MAP1LC3B* expression. We observed that the latter group of patients (bearing tumors with decreased glucose uptake and active autophagy) displayed a longer overall survival and were more sensitive to the platinum therapy compared to the ones with high *IL6R* and *SLC2A1* and expressing low levels of *MAP1LC3B* (Figure 10H,I).

## 3. Discussion

Cancer cells in the most inner part of the tumor mass experience hypoxia and nutrient shortage, due to the limited vascularization. To overcome the hostile conditions faced in the tumor microenvironment (TME), cancer cells extensively reprogram their metabolism to maximize the gain of energy [30].

Glucose is the main source of energy and intermediate metabolites for tumor mass growth. Glucose metabolism reprogramming (known as Warburg effect) consists in enhanced glycolysis, regardless the presence of oxygen, and it is considered a hallmark of cancer [31]. To fulfill the energetic demand required for cancer sustenance and progression, tumor cells consume up to 200 times more glucose than the corresponding normal cells. Oncogenes activation (e.g., *c-MYC* and *KRAS*) and tumor suppressor genes inactivation (e.g., *TP53*) in cancer cells increase the translocation of glucose transporters on plasma membrane and up-regulate the glucose intake and the glycolytic rate [32,33,34].

High amount of lactate arising from the high glycolytic rate promotes the expression of monocarboxylate transporters to remove the excess of lactate for preventing cytoplasm acidification [35,36]. This event favors the acidification of the TME, favoring tumor invasion and metastasis [37].

Ovarian cancer aggressiveness is due to its high metastatic potential that allows the tumor cells to spread out from the primary site to distant organs, giving rise to relapse and drug resistance [38]. IL-6 is one of the pro-inflammatory cytokines released either by cancer cells and or by stromal cells. IL-6 is abundant in the ascitic liquid of ovarian cancer patients, and promotes cell cycle progression and growth as well as increases the invasive phenotype of ovarian cancer [12].

Here, we report for the first time that the pro-migratory effect of IL-6 on ovarian cancer cells is suppressed if glucose is not available for triggering the glycolytic pathway, indicating that this pro-inflammatory cytokine activates its oncogenic downstream pathways only when glucose is metabolized. In the last years, the anti-cancer properties of natural compounds, along with their low toxic side effects, have attracted the interest of researchers [39]. Our group extensively reported the beneficial effects of the nutraceutical resveratrol (RV) that acts as a potent autophagy inducer, caloric restriction mimetic, and is capable to modulate the epigenome of the cancer cells, the secretion of cytokines and mitogenic factors, and to limit angiogenesis, immune suppression, and the activation of cancer-associated fibroblasts [16,17,18,19,20,27,40]. In the present work, we found that RV counteracts ovarian cancer cell migration by mimicking the phenotypic changes observed in glucose deprivation. Notably, the mechanism though which RV suppresses cancer cell motility is independent from the genetic background since similar effects were observed in OVCAR3 and OAW42 cells that differ in their mutational landscape of oncogenes and oncosuppressor genes. In detail, the addition of RV to culture medium keeps the glucose transporter GLUT1 in a perinuclear position, thus reducing the glucose intake and the glycolytic rate. As a result of glucose shortage, ovarian cancer cells increased the level of autophagy. Autophagy is deregulated in several cancers, and plays a double-edged role in the regulation of EMT [41]. In the early steps of carcinogenesis, autophagy may oppose to the acquisition of the mesenchymal phenotype by ensuring the degradation of transcription factor involved in the promotion of EMT process; on the other hand, autophagy may support the invasive potential of cancer cells by allowing their survival in the bloodstream during metastasization and participating in the selection of the more aggressive clones [42,43]. Here, we show that RV rescues autophagy along with suppression of EMT markers. This finding is in agreement with our previous work showing that inhibiting autophagy (either pharmacologically or via autophagy gene silencing) abrogated the effect of RV on EMT markers [16,19].

Cancer cells have developed several strategies to integrate metabolism and cell survival, thus providing adaptive mechanisms in response to changes in the cellular environment. The molecular link between autophagy and glycolysis is represented by hexokinase 2 (HK2). In glucose starvation, HK2 binds and sequesters mTOR, causing the detachment of the latter from the lysosome and so inducing autophagy [29]. Here, we show for the first time that RV can perform this molecular switch, indicating that this nutraceutical can mimic the beneficial effects exerted by glucose deprivation as well as the inhibition by 2-DG. Consistently, RV down-regulated the transcripts of the glycolytic metabolism and up-regulated those of several autophagy-related genes. Thus, RV acts as a “glucose restriction mimetic”, and can substitute for a glucose starvation diet or a therapeutic regimen with 2-DG.

To address the translational relevance of our in vitro findings, we conducted a bioinformatic analysis on two clinical databases. The analysis showed that ovarian cancer patients bearing a tumor with an active autophagy and a low expression of glycolytic markers had a longer overall survival and better response to platinum therapy. These findings agree with other reports from the literature, and highlight the relevance of targeting the glycolytic pathway for treating glucose-addicted cancers [3,44,45].

In conclusion, here we demonstrated the key role of the glycolytic pathway in mediating the IL-6 pro-tumorigenic activities through inhibition of autophagy, and the capability of RV to abrogate the glycolytic shift in parallel with rescuing autophagy and reprogramming ovarian cancer cells glucose metabolism toward mitochondrial respiration.

## 4. Materials and Methods

### 4.1. Cell Culture

We employed three ovarian cancer cell lines, OVCAR3 and OAW42 (SKOV3 for supplementary materials) that differ in the tumor site of origin and the genetic background (Table 1). OVCAR3 (cod. HTB-161) and SKOV3 (cod. HTB-77) cell lines were purchased from the American Type Culture Collection (ATCC) (Manassas, VA, USA). Cells were cultured in RPMI-1640 (cod. R8758; Sigma Aldrich, St. Louis, MO, USA), supplemented with 10% heat-inactivated fetal bovine serum (FBS, cod. ECS0180L; Euroclone, Milan, Italy), 1% glutamine (cod. G7513; Sigma-Aldrich, St. Louis, MO, USA) and 1% penicillin/streptomycin (PES, cod. P0781; Sigma-Aldrich, St. Louis, MO, USA). OAW42 cell line was obtained by ECACC (cod. 85073102; European Collection of Authenticated Cell Cultures; Porton Down, Salisbury, UK). OAW42 cells were cultured in minimum essential medium (MEM) (cod. M2279; Sigma-Aldrich, St. Louis, MO, USA), supplemented with 10% FBS, 1% glutamine, 1% non-essential amino acids (cod. M7145; Sigma-Aldrich, St. Louis, MO, USA), and 1% PES. Glucose deprivation assays were performed in RPMI-1640 medium without glucose (cod. R1383; Sigma-Aldrich, St. Louis, MO, USA), supplemented as previously described. All the cell lines were cultured under standard conditions (37 °C, 95 *v*/*v*% air: 5 *v*/*v*% CO_2_).

### 4.2. Reagents

IL-6 (cod. 11340066; Immunotools, Friesoythe, Germany) was dissolved in sterile water and used at a final concentration of 50 ng/mL. Resveratrol (RV, cod. R5010; Sigma-Aldrich, St. Louis, MO, USA) was dissolved in DMSO and used at a final concentration of 100 µM. Control experiments demonstrated that DMSO (final concentration: 0.01%) had no effect on cell growth and autophagy. Methylene blue (MB, cod. M.4159; Sigma-Aldrich, St. Louis, MO, USA) was dissolved in sterile water and used at a final concentration of 10 µM. 2-deoxy-D-glucose (2-DG, cod. D6134; Sigma-Aldrich, St. Louis, MO, USA) was dissolved in sterile water and used at a final concentration of 20 mM. Sodium oxamate (cod. 19057; Sigma-Aldrich, St. Louis, MO, USA) was dissolved in sterile water and used at a final concentration of 20 mM.

### 4.3. Wound Healing Migration Assay

Cells were seeded in Petri dishes at the density of 60,000 cells/cm^2^ and cultured until confluence. The cell monolayer was scratched with a sterile pipette yellow tip to produce a straight line and the debris washed out with PBS [16]. To prevent induction of autophagy, medium and treatments were renovated every 24 h. The open gap was photographed with a camera at phase contrast microscope (magnification 5×, Zeiss AXIOVERT 40CFL, Zeiss, Oberkochen, Germany) at the indicated times. The rate of healing was estimated by ImageJ software based on the area free of cells as previously reported [16]. Data are calculated for three different fields per each condition.

### 4.4. Immunofluorescence

Cells were seeded onto sterile coverslips at a density of 25,000–35,000 cells/cm^2^ and allowed to adhere. A scratch was performed on each coverslip, using a pipette tip, before the treatments. At the end of the experiment, coverslips were fixed, permeabilized and stained as previously described in [17].

Coverslips were mounted onto glasses using SlowFade reagent (cod. S36936; Life Technologies, Paisley, UK) and imaged at fluorescence microscope (Leica Microsystems DMI6000, Wetzlar, Germany).

### 4.5. Glucose Uptake Assay

Cells were seeded onto coverslips at a density of 25,000–35,000 cells/cm^2^ and allowed to adhere. A scratch was performed on each coverslip, using a pipette tip, before the treatments. One hour before the time-point tested, 50 μM 2-(N-(7-nitrobenz-2-oxa-1,3-diazol-4-yl)Amino)-2-deoxy-D-glucose (2-NBDG, cod. N13195; Life Technologies, Paisley, UK) was added and incubated at 37 °C for 1 h. Therefore, coverslips were washed three times with PBS, mounted onto glasses and acquired immediately at fluorescence microscope (Leica Microsystems DMI6000, Wetzlar, Germany).

### 4.6. Anion Superoxide Production by MitoSOX™ Fluorescence Assay

Cells were seeded onto coverslips at a density of 25,000–35,000 cells/cm^2^ and allowed to adhere. A scratch was performed on each coverslip using a pipette tip before the treatments. Mitochondrial anion superoxide was detected on living cells by using 5 μM MitoSOX™ red staining (cod. M36008; Life Technologies, Paisley, UK) as previously described in [46]. Coverslips were washed three times with PBS, mounted onto glasses, and images were acquired immediately using a fluorescence microscope (Leica Microsystems DMI6000, Wetzlar, Germany)

### 4.7. Mitochondria Viability by Cell Tracker™ Blue Fluorescence Assay

Cells were seeded onto coverslips at a density of 25,000–35,000 cells/cm^2^ and allowed to adhere. A scratch was performed on each coverslip using a pipette tip before the treatments. To monitor mitochondrial functionality, cells were stained with the fluorescent dye 5 μM Cell Tracker™ (Cell Tracker Blue-CMAC 7-amino-4-chloromethylcoumarin, cod. C2110; Life Technologies, Paisley, UK) as previously described in [47]. Coverslips were washed three times with PBS, mounted onto glasses, and images were acquired immediately using a fluorescence microscope (Leica Microsystems DMI6000, Wetzlar, Germany).

### 4.8. Western Blot Analysis

Cells were seeded on Petri dishes at a density of 40,000–50,000 cells/cm^2^ and treated as indicated when confluence reached approximately 80%. Cell homogenates were prepared by freeze-thawing and ultrasonication in NaDOC lysis buffer containing protease inhibitors as described in [27]. The blots were detected using enhanced chemiluminescence reagents (ECL, cod. NEL105001EA; Perkin Elmer, Waltham, MA, USA) and developed with the ChemiDoc XRS instrument (BioRad, Hercules, CA, USA). Intensity of the bands was estimated by using Quantity One Software 4.5.0 (BioRad, Hercules, CA, USA). Data were reproduced at least three times separately.

### 4.9. Antibodies

The following primary antibodies were used for either immunofluorescence or Western blotting: rabbit anti-GLUT1 (1:50, cod. 07-1401; Millipore, Burlington, MA, USA); rabbit anti-mTOR (1:50, cod. 2983; Cell Signaling, Danvers, MA, USA); mouse anti-HK2 (1:100, cod. NBP2-02272; Novus Biologicals, Milan, Italy); mouse anti-LAMP1 (1:1000, cod. 555798; BD Biosciences, Franklin Lakes, NJ, USA); rabbit anti-LC3 (1:1000, cod. L7543; Sigma-Aldrich, St. Louis, MO, USA); mouse anti-N-cadherin (1:50, cod. 610920; BD Biosciences, Franklin Lakes, NJ, USA); mouse anti-E-cadherin (1:50, cod. 610404; BD Biosciences, Franklin Lakes, NJ, USA); rabbit anti-TWIST1 (1:1000, cod. T6451; Sigma Aldrich, St. Louis, MO, USA); mouse anti-β-Tubulin (1:1000, cod. T5201; Sigma-Aldrich, St. Louis, MO, USA); mouse anti-IL-6R (1:1000, cod. AHR0061; Invitrogen, Waltham, MA, USA); mouse anti-β-Actin (1:2000, cod. A5441; Sigma-Aldrich, St. Louis, MO, USA).

### 4.10. One Color Microarray Genome-Wide Gene Expression Analysis

OVCAR3 cells were seeded on Petri dishes at a density of 40,000–50,000 cells/cm^2^. When confluence reached approximately 80%, cell monolayer was treated with 100 µM resveratrol for 24 h. Total RNA was isolated from the cells using absolutely RNA mRNA kit (Agilent Technologies, Palo Alto, CA, USA) and samples were processed as previously reported in [16]. Raw data elaboration was carried out with Bioconductor (www.bioconductor.org, accessed on 1 March 2019) [48], using R statistical language. Background correction was performed with the normexp method and quantile was used for between-array normalization. The linear models for microarray analysis (LIMMA) package was then used to identify differentially expressed genes between the different experimental conditions. The empirical Bayes method was used to compute the moderated t-statistics [49]. Transcripts with a log base two-fold change (logFC) greater than +0.20 or lower than −0.20 were considered as differentially expressed.

### 4.11. Bioinformatic Analysis

Kaplan–Meier curves and correlation studies were conducted by extracting clinical data from The Cancer Genome Atlas (TCGA) database (www.cBioportal.org, last accessed on 25 May 2022) [50]. The analysis was conducted on an ovarian serous cystadenocarcinoma dataset (TCGA Nature 2011, accounting for 316 patients). TCGA gene expression profile was measured using the Illumina HiSeq 2000 RNA Sequencing platform (Illumina Inc., 9885 Towne Centre Drive, San Diego, CA 92121, USA). RSEM (RNA-Seq by Expectation-Maximization) normalized count was used as gene level expression. Gene variables were measured by median absolute deviation (MAD). mRNA expression and clinical information including TP53 status and overall survival were downloaded from the cBioportal.org. Patients were grouped based on the level of mRNA expression, low versus high groups were defined relative to the median expression level of overall patient cohort. To reduce the potential bias from dichotomization, the mRNA expression of relevant biomarkers was compared based on high and low expression-based groups using t-test (Welch two Sample *t*-test) by R. All cut-off values were set before the analysis, and all the tests were two-tailed. Survival curves were obtained by using the Kaplan–Meier plotter (www./kmplot.com/, last accessed on 25 January 2022) and the analysis was conducted on a larger ovarian patients’ dataset (comprising only in TP53 mutated cases—506 samples) to corroborate the findings achieved from TCGA interrogation. The correlation between the mRNA expression of relevant biomarkers and the response to platinum therapy is represented in histograms. These graphs report the number of patients with a specific transcriptome profile that were classified as resistant (disease progression within six months from primary therapy), sensitive (relapse or disease progression at least six months after the end of treatment), or too-early resistant (resistance occurs as soon as treatment start) based on the clinical outcome reported in the database. All statistical analyses were performed by R (3.6.1 version, The R Foundation for Statistical Computing, Vienna, Austria) and SAS software (9.4. version, SAS Institute Inc., Cary, NC, USA) using SAS/STATs procedures.

### 4.12. Statistical Analysis

All data refer to at least three separate experiments. Data in histograms are shown as average  ±  S.D. Statistical analysis was performed with GraphPad Prism 5.0 software. Bonferroni’s multiple comparison test after one-way ANOVA analysis (unpaired, two-tailed) was employed. Significance was considered as follow: **** *p*< 0.0001; *** *p* < 0.001; ** *p* < 0.01; * *p* < 0.05.

## 5. Conclusions

The present work demonstrates, for the first time, that ovarian cancer cells migration relies on IL-6-induced aerobic glycolysis. Here we also show that resveratrol acts as a “glucose restriction mimetic” that prevents mitochondrial oxidation and induces autophagy to limit cancer cell migration.

Recapitulating these in vitro findings, which are of clinical relevance, the bioinformatic analysis conducted on TCGA data shows that patients exhibiting a downregulation of glycolytic marker and of IL-6R along with markers of active autophagy display a better prognosis.

Chemoresistance and side effects limit the efficacy of the current standard therapeutic regimens. Therefore, there is an urgent unmet need to identify alternative strategies that may improve patients’ clinical outcome, by sensitized tumor cells to therapy, and to limit secondary metastasis and relapses, as well as reducing unwanted adverse effects. A therapeutic approach including RV is very attractive due to its pleiotropic effects that allow to concomitantly ameliorate multiple aspects of the malignant phenotype of cancer cells. Our data support the view that RV can contrast ovarian cancer progression by hampering the glucose metabolism and limiting the risk of metastasis, supporting the use of this natural compound as an adjuvant for conventional anti-cancer therapy in highly aggressive glucose-addicted ovarian cancers.

## Figures and Tables

**Table 1:** Genetic background of ovarian cancer cell lines. OVCAR3 cell line originates from the malignant ascites of ovary epithelial carcinoma. OAW42 cell line derives from the ascites of ovary mucinous cystoadenocarcinoma. SKOV3 cell line arises from the ascites of ovary adenocarcinoma.

	*TP53*	*PTEN*	*PI3KCA*
**OVCAR3**	R248Q	Null	Wild-type
**OAW42**	Wild-type	Wild-type (methylated *)	H1047L
**SKOV3**	Null	Wild-type	Wild-type

* As published in [21].

## Data Availability

Statement: the data that support the findings of this study are available from the corresponding author upon reasonable request.

## Supplementary Materials

The supporting information can be downloaded at: https://www.mdpi.com/article/10.3390/ijms24021723/s1.
