# Supplementary material for: Glycolysis Inhibition of Autophagy Drives Malignancy in Ovarian Cancer: Exacerbation by IL-6 and Attenuation by Resveratrol
Source: Int J Mol Sci. 2023 Jan 15;24(2):1723. doi: 10.3390/ijms24021723 (PMC9866176; doi:10.3390/ijms24021723)
Supplement: Supplementary file 1 [file ijms-24-01723-s001.zip › ijms-2139369-supplementary.pdf]

S1

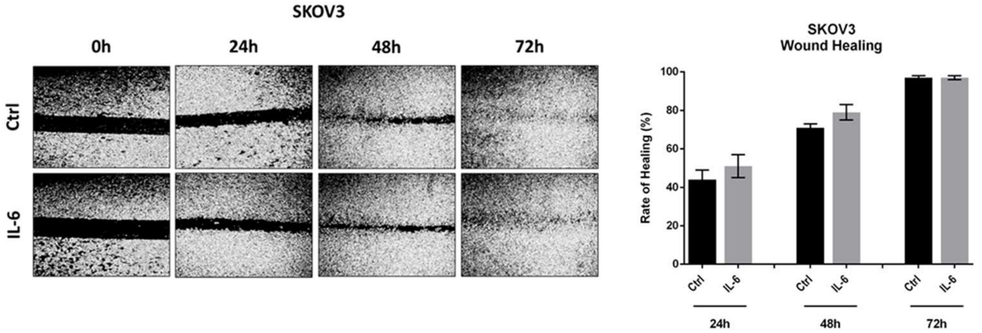

Figure S1. IL-6 fails to stimulate SKOV3 cell migration. SKOV3 cells were exposed or not to 50 ng/ml IL-6. Medium was renewed every 24 hours. The wound closure was monitored up to 72 hours by using a phase-contrast microscope. The rate of healing (%) for each experimental time-point, indicated as the distance between the margins of the wound, was calculated by using ImageJ software. Data represent the average ± SD calculated for three different fields per each condition.

S2

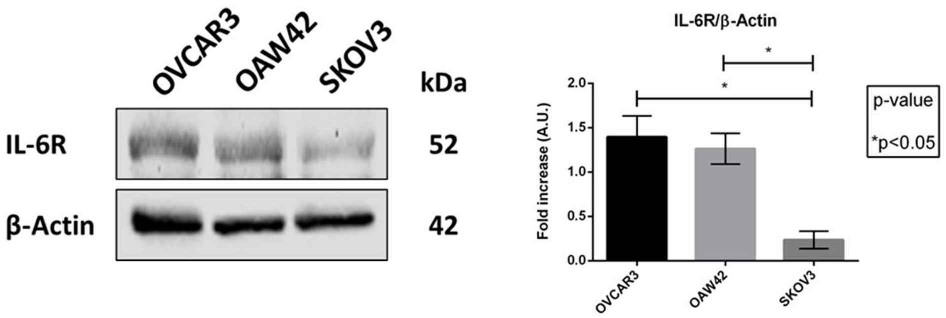

Figure S2. Expression of IL-6R in the three ovarian cancer cell models. OVCAR3, OAW42, and SKOV3 cell homogenates were analyzed by western blotting for the expression of IL-6R. The filter was probed with β-Actin as loading control. The western blotting is representative of three replicates and densitometric data are reported in the graph.
